# Salivary Biomarkers in Pediatric Acute Appendicitis: Current Evidence and Future Directions

**DOI:** 10.3390/children12101342

**Published:** 2025-10-06

**Authors:** Zenon Pogorelić, Miro Jukić, Tomislav Žuvela, Klaudio Pjer Milunović, Ivan Maleš, Ivan Lovrinčević, Jasenka Kraljević

**Affiliations:** 1 Department of Pediatric Surgery, University Hospital of Split, 21000 Split, Croatia; 2 Department of Surgery, School of Medicine, University of Split, 21000 Split, Croatia; 3 Department of Surgery, University Hospital of Split, 21000 Split, Croatia

**Keywords:** acute appendicitis, saliva, biomarkers, salivary biomarkers, children

## Abstract

Background: Acute appendicitis is the most common surgical emergency in children, yet timely and accurate diagnosis remains challenging due to nonspecific clinical presentations and limitations of imaging and blood tests. Saliva has emerged as a promising diagnostic medium because it is non-invasive, painless, inexpensive, and highly acceptable for pediatric patients. Salivary biomarkers may provide rapid and child-friendly adjuncts to existing diagnostic pathways. Methods: A systematic literature search was performed in Ovid/MEDLINE, Scopus, Web of Science, and the Cochrane Library to identify studies assessing salivary biomarkers in pediatric appendicitis. Eligible studies included children with suspected or confirmed appendicitis and evaluated the diagnostic accuracy of salivary markers compared to clinical, laboratory, or imaging standards. Results: To date, only three salivary biomarkers have been investigated. Leucine-rich α-2-glycoprotein 1 (LRG1) demonstrated high specificity of 100% but low sensitivity of 35–36%, with diagnostic accuracy ranging from AUC 0.77 to 0.85. C-reactive protein (CRP) showed excellent diagnostic performance with sensitivity of 91.3% and specificity of 95.4% (AUC 0.97), and strong correlation with serum CRP (ρ = 0.96). Irisin showed sensitivity of 90% and specificity of 60% with estimated AUC around 0.75, suggesting potential as an adjunct marker but limited as a standalone test. Conclusions: Salivary biomarkers in pediatric appendicitis are promising but remain underexplored, with evidence limited to small, single-center studies totalling fewer than 300 patients. Their advantages include feasibility, tolerability, and suitability for integration into point-of-care testing. Future research should focus on multicenter validation, development of multi-marker salivary panels, and application of biosensor technologies. With further evidence, salivary diagnostics could complement existing strategies and improve the accuracy and child-friendliness of appendicitis care.

## 1. Introduction

Acute appendicitis is a common reason for emergency abdominal surgery in children. Current diagnostic approaches favor ultrasound as the initial test, with computed tomography (CT) or magnetic resonance imaging (MRI) used for unclear cases [1]. International guidelines emphasize that appendicitis is a time-sensitive condition, where quick diagnosis and efficient use of resources directly affect outcomes [2]. Population-based studies have refined intraoperative severity criteria, providing standardized benchmarks for assessing outcomes and testing novel diagnostic approaches [3]. Evidence on optimizing care, such as trials of postoperative antibiotics in non-perforated cases, underscores that accurate early diagnosis leads to safer, more effective management [4].

Many pediatric centers increasingly use MRI when resources allow, aiming to reduce radiation exposure without losing diagnostic accuracy [5]. Rapid MRI protocols now provide reliable results in emergencies, avoiding ionizing radiation [6]. Pathways that escalate to MRI after inconclusive ultrasound help address operator dependency and non-visualized appendices, enabling faster, safer decisions [7]. However, imaging alone is not always definitive. Systematic reviews show that clinical scoring systems, often used alongside imaging, perform inconsistently at the bedside and highlight the need for additional data in uncertain cases [8]. Tools like the Pediatric Appendicitis Risk Calculator (pARC) remain useful but work best when combined with other inputs to improve accuracy across pediatric populations [9].

Experts in infectious disease, emergency medicine, and radiology support structured imaging pathways beginning with ultrasound and reserving CT or MRI for unresolved cases [10]. Global epidemiology shows wide variation in incidence and perforation rates, emphasizing that pre-test probability depends on setting and patient group [11]. Large multicentre studies link diagnostic delays to higher perforation risk in younger children, highlighting the need for rapid, reliable assessment [12]. Yet operator dependence and nondiagnostic ultrasounds remain common, especially in busy or resource-limited settings, supporting the search for non-invasive, child-friendly adjuncts [11,13,14].

These challenges underscore the need for diagnostics that are quick, reliable, and well tolerated by children, while feasible during short observation periods and easily integrated into existing clinical pathways. Saliva has emerged as a promising candidate: collection is painless, inexpensive, and highly accepted by children. Analytically, saliva contains proteins, nucleic acids, extracellular vesicles, and metabolites reflecting local and systemic inflammation relevant to appendicitis pathobiology [14]. Reviews from oral biology and analytical sciences highlight opportunities and challenges, timing, stimulation, handling, and storage that must be standardized for reproducible clinical results [15]. Public health initiatives have shown saliva performs well for accurate, large-scale screening with standardized handling, supporting its potential role in pediatric diagnostics beyond infectious disease [16]. Additional reviews detail assay technologies, validation requirements, and clinical translation, offering insights especially suited to emergency settings needing speed and tolerability [17].

Recent technological advances have brought saliva testing closer to routine use. Portable point-of-care devices, including advanced lateral-flow and electrochemical systems, can now deliver accurate results in minutes at the bedside without a lab [18]. Emerging tools like auto-signal-enhanced immunoassays are further increasing sensitivity and reliability, bridging the gap between research and real-world pediatric emergency care [19].

In appendicitis, early research on salivary biomarkers is promising. The most studied biomarker in children is LRG1, with preliminary urinary findings supporting its biological rationale. C-reactive protein (CRP) also remains a plausible target, correlating with appendicitis severity. Irisin is a myokine secreted by skeletal muscle through cleavage of the FNDC5 protein. While initially studied in the context of energy homeostasis and metabolism, it has more recently been implicated in systemic inflammatory processes, making it a plausible salivary biomarker candidate for acute appendicitis [20,21,22,23,24,25,26,27,28,29,30].

## 2. Methods

### 2.1. Literature Search and Study Selection

**Figure 1:**
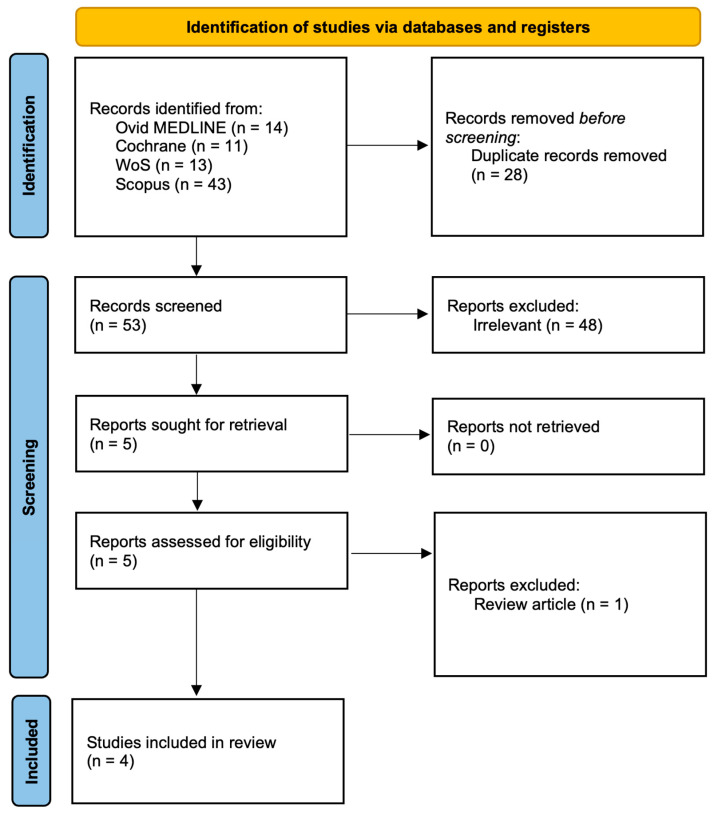
PRISMA flow diagram. MEDLINE—Medical Literature Analysis and Retrieval System Online; WoS—Web of Science.

We performed a comprehensive literature search in Ovid MEDLINE, Scopus, Web of Science, and the Cochrane Library to identify studies investigating salivary biomarkers in pediatric acute appendicitis. The last search was conducted on 17 September 2025. The search combined MeSH terms and free-text terms related to saliva/salivary, appendicitis/appendectomy, and pediatric populations (including children, adolescents, infants, and pediatric/paediatric terminology) incorporating Boolean operators (AND, OR) to refine the results. Searches were limited to the English language but not limited regarding date restrictions. Titles and abstracts were independently screened by two reviewers (Z.P., I.M.) according to predefined eligibility criteria. Studies were included if they: (i) involved patients under 18 years of age, (ii) assessed salivary biomarkers in the context of suspected or confirmed acute appendicitis, and (iii) reported original clinical data. Additionally, reference lists of all included studies and relevant reviews were screened manually to identify further eligible publications (Figure 1).

### 2.2. Data Extraction and Analysis

From each included study, we extracted data on study design, population characteristics, sample size, biomarker investigated, collection method, and analytical assay. Reported diagnostic performance metrics, including sensitivity, specificity, and area under the receiver operating characteristic (ROC) curve (AUC), were recorded as presented by the original authors without unit conversion since biomarkers were expressed in different concentrations (e.g., ng/mL, mg/L) across studies. Information on cutoff values, statistical significance, and correlation with serum levels was also collected when available. Methods of saliva collection, such as passive drool or swab-based techniques, were noted but not standardized for comparative analysis, as the primary aim was to summarize all available evidence regardless of collection approach. When multiple outcomes were reported, those most relevant to diagnostic accuracy were prioritized. Given these methodological differences, results were synthesized descriptively rather than pooled. Due to the limited number of studies and their methodological heterogeneity, no formal meta-analysis was conducted; instead, results were synthesized narratively and summarized in tabular format to allow descriptive comparison.

Extracted metrics were tabulated to allow comparison across studies. All figures and summary plots were generated using Python (version 3.10) with the Matplotlib library (version 3.7).

## 3. Saliva as a Diagnostic Medium

### 3.1. Physiology and Composition of Saliva

Saliva is mainly produced by the parotid, submandibular, sublingual, and minor glands. It is about 99% water, with the rest composed of electrolytes, proteins, enzymes, mucins, antimicrobial agents, and other organic/inorganic molecules [31,32,33].

Saliva secretion is a nerve-mediated reflex regulated by both parasympathetic and sympathetic systems. Parasympathetic activity increases watery secretion, while sympathetic stimulation yields smaller but protein-rich volumes [34,35,36]. Acinar cells secrete isotonic fluid, later modified in ducts through ion reabsorption and secretion, resulting in hypotonic saliva [31,33,37]. Secretion is stimulated by food, taste, and chewing, and inhibited by anxiety or dehydration [34,37]. Flow ranges from 0.3–0.4 mL/min unstimulated to 1.5–2.0 mL/min when stimulated, with a total of 0.5–1.5 L/day [37].

Major components include water, electrolytes, bicarbonate, phosphate, proteins and enzymes (α-amylase, lingual lipase, mucins, lysozymes, lactoferrin, statherin), immunoglobulins (mainly IgA), organic molecules (urea, glucose, peptides), and cells (epithelial, leukocytes) [32,33,37]. Saliva lubricates tissues, aids chewing and swallowing, supports taste, protects against pathogens, buffers acids, remineralizes teeth, and initiates starch digestion [37,38].

Composition depends on gland origin, flow, and stimulation [37,39]. Parotid saliva is serous and enzyme-rich, submandibular mixed, and sublingual mainly mucous [31,37,39]. Variations also reflect circadian rhythms, hydration, medications, disease, and age [31,34]. Unstimulated saliva is rich in bicarbonate and mucins, while stimulated saliva contains more electrolytes and enzymes like amylase [37].

### 3.2. Advantages over Serum/Urine

Saliva offers several key advantages over serum and urine for diagnostic and monitoring purposes: it can be collected non-invasively, repeatedly, at lower cost, and with minimal training [40,41,42].

Saliva collection is rapid, safe, and can be repeated frequently, which is a significant advantage in monitoring circadian or disease-related fluctuations [40,43]. It is also generally easier and less costly to handle, transport, and store than serum or urine, and saliva does not clot, simplifying laboratory processing [40,42]. Many substances found in blood are also present in saliva, making it functionally equivalent in many diagnostic respects [40,41]. These benefits increase patient compliance, reduce anxiety, lower overall test costs, and are particularly important in resource-limited settings or mobile testing programs [40,41,42,44].

However, for certain substances or hormones, the sensitivity and specificity of saliva can differ from serum or urine; test selection should consider the clinical context and available assay validation [43,44].

### 3.3. Challenges and Limitations of Saliva as a Diagnostic Medium

Salivary flow rate shows wide variation depending on hydration status, circadian rhythms, emotional state (stress, anxiety), medications (e.g., anticholinergics, antidepressants), disease states (e.g., Sjögren’s syndrome, dehydration), age, and even posture during collection. Unstimulated flow may be very low in some individuals, while stimulated saliva can vary greatly with chewing or taste stimuli. Such variability can alter analyte concentrations through dilution, complicating standardization and quantitative comparison. Very low flow or xerostomia can severely compromise collection, especially in elderly or medically compromised patients [37,39,45,46].

Sample handling also presents challenges. Saliva is prone to enzymatic degradation and bacterial growth if not promptly processed or stored. Proteins, hormones, and nucleic acids may degrade rapidly at room temperature, risking inaccurate results if delays occur. Samples may also be contaminated by food, drink, blood, or environmental substances.

Furthermore, the choice of collection method (passive drool, swabs, absorbent devices) influences analyte recovery and introduces variability. Finally, the lack of standardized collection and handling protocols, compared with blood or urine, limits reproducibility and comparability across studies and clinical settings [45,46].

### 3.4. Diagnostic Use of Saliva in Other Pediatric Conditions

Saliva is increasingly recognized as a valuable diagnostic tool in pediatrics for viral infections, stress response, and inflammatory markers such as CRP. It enables detection of both direct (viral DNA/RNA) and indirect (antibody, protein) biomarkers. This allows non-invasive diagnosis of herpesviruses, human papillomavirus, polyomaviruses, torque teno virus, SARS-CoV-2, mumps, measles, HIV, hepatitis viruses, and Zika virus [47,48,49]. For COVID-19 in children, saliva is reliable for both symptomatic and asymptomatic cases, enabling detection of viral RNA and immune response (IgA, IgG, IgM). Its ease, safety, and suitability for repeated or parent-supervised collection make it practical for mass screenings and serial monitoring [48,49].

Salivary cortisol and alpha-amylase are established non-invasive markers of physiological and psychological stress. Cortisol reflects hypothalamic–pituitary–adrenal (HPA) axis activity, while alpha-amylase indicates sympathetic activation, responding rapidly to acute and chronic stress. They can be used to track stress, anxiety, pain response, and therapy outcomes in both healthy and medically complex children [50,51,52].

Salivary CRP correlates well with serum CRP in children [53,54]. It is elevated in pneumonia and sepsis, correlating with severity and recovery, making it useful for monitoring acute infections when blood sampling is difficult. Particularly in neonates, it offers a non-invasive alternative to repeated blood draws [53,54,55]. Saliva can also be used for oxidative stress markers and immunological monitoring (salivary IgA, IgM, IgG for vaccine responses and mucosal immunity) [50,54,55].

## 4. Current Evidence on Salivary Biomarkers in Pediatric Appendicitis

Although still in their early stages, several pilot studies have investigated the potential of salivary biomarkers for diagnosing acute appendicitis in children. The clinical signs of appendicitis in pediatric patients are often non-specific, and traditional diagnostic methods such as clinical scoring systems, laboratory tests (e.g., WBC, CRP), and imaging techniques can be limited by factors like availability, cost, or accuracy, especially in early or atypical cases. In this context, saliva has become an appealing diagnostic medium due to its non-invasive collection, ease of handling, and increasing evidence that it reflects systemic inflammatory responses through the transudation of plasma proteins and the local secretion of cytokines.

The most extensively studied targets to date include leucine-rich α-2-glycoprotein 1 (LRG1), C-reactive protein (CRP), and irisin. Each of these biomarkers is known to be involved in systemic inflammatory conditions and has been previously validated in serum or urine for diagnosing appendicitis. Detecting them in saliva is a new and important development, especially in pediatrics, where reducing patient discomfort and making diagnostics simpler are essential. These studies indicate a promising diagnostic potential, especially in situations where non-invasive methods are preferred and quick triage or early screening can improve management and lower the risk of perforation or unnecessary surgery. Key findings from all studies examining salivary biomarkers in pediatric appendicitis are summarized in Table 1.

### 4.1. LRG1 (Leucine-Rich α-2-Glycoprotein 1)

A pilot study by Yap et al. evaluated salivary LRG1 in 34 children aged 4–16 years (17 with histologically confirmed appendicitis, 17 controls). Saliva was collected using the SalivaBio Children’s Swab, and LRG1 was measured by ELISA (IBL International). The median salivary LRG1 level was significantly higher in children with appendicitis (0.294 ng/μg of total salivary protein) compared to controls (0.126 ng/μg; *p* = 0.008). At a threshold of 0.33 ng/μg (which equals roughly 330–495 ng/mL, depending on total protein concentration), the test had a specificity of 100% and a sensitivity of 35.3%, with an AUC of 0.77. Additionally, LRG1 levels were significantly higher in patients with perforated appendicitis than in those with uncomplicated disease (*p* = 0.05). These findings suggest that LRG1 has strong rule-in potential, especially at higher concentrations, though its low sensitivity limits its use as a standalone test [56].

A more recent and larger study by Tintor et al. involved 92 pediatric patients presenting with abdominal pain (46 with histologically confirmed acute appendicitis and 46 controls). Salivary samples were collected using passive drool and analyzed with ELISA (R&D Systems). The median LRG1 concentration was significantly higher in children with appendicitis (233.5 ng/mL; IQR 174.8–368.4) compared to controls (55.9 ng/mL; IQR 36.5–95.6), with *p* < 0.0001. The optimal diagnostic threshold was identified as 352.6 ng/mL, giving a specificity of 100% and a sensitivity of 36.0%. ROC analysis showed good discriminatory ability, with an AUC of 0.851 (95% CI: 0.778–0.924). Although the test had high specificity, which is especially useful for confirming diagnosis, it had moderate sensitivity, limiting its effectiveness in ruling out appendicitis. Interestingly, there was no statistically significant difference in LRG1 levels between patients with uncomplicated and complicated appendicitis, indicating its main role may be in initial detection rather than staging. These findings confirm the diagnostic potential of salivary LRG1 and support the results from the earlier pilot by Yap et al., with a larger sample size and more robust statistical validation [21].

### 4.2. Irisin

In a prospective study, Bakal et al. enrolled 60 children and categorized them into groups with appendicitis, non-specific abdominal pain, and healthy controls. Saliva samples were collected using non-stimulated passive drool and analyzed with ELISA (Sunred Biological Technology, Shanghai, China). The results indicated that salivary irisin levels were significantly higher in children with appendicitis (mean 29.4 ± 5.1 ng/mL) compared to healthy controls (18.5 ± 6.3 ng/mL), with a reported *p* value < 0.001. At a cutoff value of 19.6 ng/mL, the sensitivity and specificity for diagnosing appendicitis based on salivary irisin were 90% and 60%, respectively. Although ROC curves were not visually shown, the authors interpreted these values as indicative of a promising diagnostic profile. Furthermore, salivary irisin levels showed a moderate correlation with serum and urinary irisin concentrations (r = 0.36–0.39; *p* < 0.05), suggesting that salivary levels may reflect systemic inflammatory status. Irisin, a myokine initially linked to metabolic regulation and energy homeostasis, has recently been associated with inflammatory processes. Its elevation in appendicitis likely indicates neutrophil activation and immune response modulation. However, the small sample size, lack of multivariable analysis, and absence of a well-defined clinical comparator group limit the generalizability of these findings. Additionally, the relatively low specificity suggests a risk of false-positive results, especially in other inflammatory or infectious conditions. Nonetheless, this study supports the potential of irisin as a salivary biomarker candidate for appendicitis and provides a foundation for future larger-scale validation studies [57].

### 4.3. CRP (C-Reactive Protein)

The first study to assess salivary CRP in pediatric appendicitis was recently conducted by Milunović et al., involving 89 children (46 with appendicitis and 43 controls). Saliva was collected through passive drool and analyzed using ELISA (Salimetrics). The median CRP level in saliva was significantly higher in the appendicitis group (35.7 mg/L) compared to controls (1.1 mg/L), with *p* < 0.001. ROC analysis showed excellent diagnostic accuracy, with an AUC of 0.97. Using a cutoff value of 6.95 mg/L, the test achieved 91.3% sensitivity and 95.4% specificity. Salivary CRP also correlated strongly with serum CRP (Spearman ρ = 0.963, *p* < 0.001), and Bland–Altman analysis confirmed agreement between the two measurements, with acceptable variation in most cases. These findings suggest that salivary CRP reflects serum CRP in both diagnostic performance and concentration trends, supporting its potential for inclusion in non-invasive diagnostic methods, including point-of-care or rapid tests [58].

### 4.4. Comparative Diagnostic Performance of Salivary Biomarkers

The diagnostic accuracy of the three salivary biomarkers investigated to date: LRG1, irisin, and CRP shows marked variation in their clinical utility. Salivary CRP demonstrated the most robust performance, with an AUC of 0.97 and both sensitivity (91.3%) and specificity (95.4%) above 90%. This places CRP as the most promising candidate for non-invasive diagnosis, particularly because of its strong correlation with serum CRP (ρ = 0.96, *p* < 0.001) reported in the original study. By contrast, LRG1, although highly specific (100% in both published studies), showed poor sensitivity (35–36%), limiting its role as a screening or rule-out tool. Its strength lies in confirming the diagnosis when elevated, but many true cases would be missed if it were used alone. Irisin occupies an intermediate position, with high sensitivity (90%) but relatively low specificity (60%), suggesting that it may be prone to false-positive results in the presence of other inflammatory conditions.

**Figure 2:**
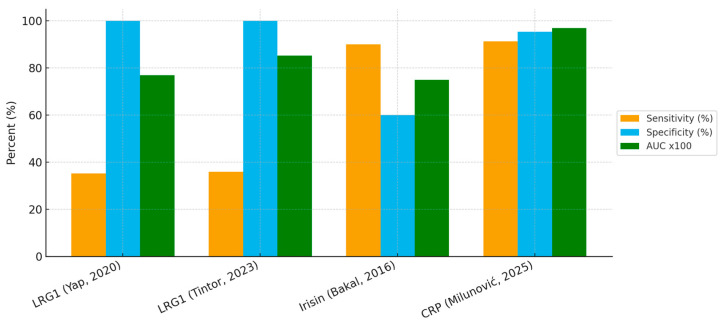
Diagnostic performance of salivary biomarkers in pediatric appendicitis [21,56,57,58].

When visualized together, the comparative bar chart (Figure 2) highlights these trade-offs between sensitivity and specificity across biomarkers.

**Figure 3:**
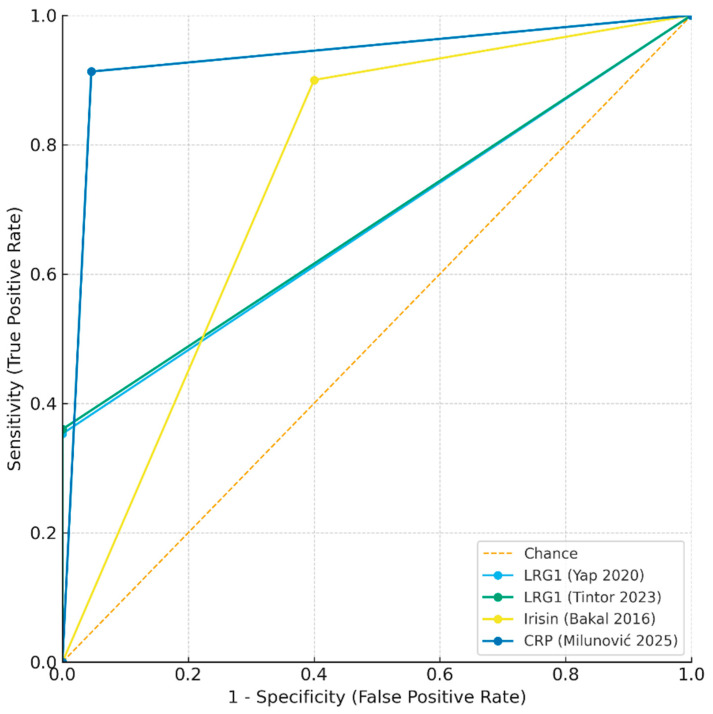
Approximate receiver operating characteristic (ROC) curves for salivary biomarkers in pediatric appendicitis [21,56,57,58].

The approximate ROC curves (Figure 3) demonstrate the superior overall discriminatory ability of CRP compared with LRG1 and irisin. Collectively, these findings suggest that single-marker strategies may be insufficient, and that future work should prioritize multi-marker salivary panels, potentially combining the high specificity of LRG1 with the high sensitivity of CRP or irisin, to maximize diagnostic performance.

## 5. Comparison with Serum and Urinary Biomarkers

In recent years, the search for reliable biomarkers of acute appendicitis has expanded from conventional serum parameters to novel non-invasive alternatives such as urinary and salivary markers. Serum remains the most widely studied biological fluid, with numerous established and emerging proteins used to support diagnosis and severity assessment. However, as invasive blood collection can be distressing in children, interest in saliva-based markers is increasing, making comparison with serum particularly relevant.

### 5.1. Serum Markers

Various cells secrete immunomodulatory proteins can exert various signalling and defence functions during the inflammatory cascade [59,60,61]. Nowadays, numerous serum biomarkers are used for acute appendicitis; here we provide a brief overview of the most commonly used ones. In comparison, salivary biomarkers are less established but offer the advantage of being non-invasive and easier to obtain in children.

#### 5.1.1. C-Reactive Protein

CRP is an acute-phase reactant that shows elevated levels 8–10 h after the onset of inflammatory processes [60]. CRP serves as an indicator of advanced appendicitis rather than an early diagnosis of simple appendicitis, and of advanced inflammation as opposed to explicit appendicitis [62,63]. Studies have described in detail that elevated CRP levels may correspond to further complications of appendicitis, e.g., perforation or abscess [64]. Despite the fact that there is some evidence that CRP may be elevated in appendicitis, the current test qualities are not high enough to be used as an independent diagnostic test. In addition, CRP is also regularly elevated in a variety of diseases, making it difficult to differentiate appendicitis from other diseases in children. For this reason, the Canadian Association of Paediatric Surgeons has issued a “Picking Carefully” proclamation advising against regularly obtaining CRP levels in children with suspected appendicitis, as this is considered unnecessary and does not influence the physician’s diagnosis [65]. Interestingly, salivary CRP has also been investigated and shows good correlation with serum levels, suggesting saliva might provide a less invasive alternative for monitoring systemic inflammation in appendicitis [58].

#### 5.1.2. Procalcitonin (PCT)

Another protein marker is PCT, which is released due to bacterial infections and, in some clinical situations, can be clearly linked to the severity and extent of bacterial disease [61]. Ongoing research suggests that PCT is likely to have diagnostic value for acute appendicitis in the pediatric population [66]. Notably, PCT has been found to have both higher sensitivity (97%) and specificity (80%) for diagnosing appendicitis than CRP (95% and 74%, respectively) [67]. The PCT level increases with the severity of infection in children and could therefore serve as a useful marker to differentiate between uncomplicated and complicated appendicitis [68].

#### 5.1.3. Bilirubin

Bilirubin is a product of red blood cell breakdown and has also been recommended as a specific marker to support the diagnosis of complicated appendicitis in children [69]. Increased total bilirubin levels in the serum can serve as an indicator of perforated appendicitis in children. The serum bilirubin level is an inexpensive, simple, and readily available laboratory marker and should therefore be recommended in the initial investigation for acute appendicitis in pediatric patients [70].

#### 5.1.4. Interleukine-6 (IL-6)

IL-6 is a basic proinflammatory cytokine released during inflammatory processes, such as bacterial invasion of the appendix and the subsequent recruitment of neutrophils [71]. Excessive and sustained production of IL-6 has been linked to various inflammatory conditions, including rheumatoid joint pain, systemic lupus erythematosus, and coronary artery disease [72].

Although IL-6 is considered an inflammatory marker in appendicitis, it does not appear to provide a significant diagnostic advantage over traditional markers like WBC and CRP [73]. While its use solely for diagnosis has been discussed, IL-6 could still serve as an effective predictive biomarker for distinguishing between patients with acute uncomplicated appendicitis and those with complicated appendicitis [74]. Early evidence indicates that IL-6 can also be detected in saliva, though its diagnostic performance in appendicitis is less well studied than in serum.

#### 5.1.5. Hyponatraemia

Hyponatremia is a new laboratory marker linked to complicated appendicitis and may have better predictive power than previously established predictors of appendiceal perforation. The exact cause of hyponatremia in patients with complicated appendicitis is unknown but is probably mediated by antidiuretic hormone [75,76]. Recent meta-analysis has confirmed that hyponatremia in pediatric patients may be a clear sign of complicated appendicitis [77].

#### 5.1.6. Leucine-Rich Alpha-2-Glycoprotein 1 (LRG1)

Leucine-rich alpha-2-glycoprotein 1 (LRG1) has recently emerged as a promising serum biomarker for diagnosing acute appendicitis. LRG1 is an acute-phase protein mainly secreted by hepatocytes and neutrophils in response to inflammatory stimuli. Its expression is increased by pro-inflammatory cytokines such as interleukin-6, which are abundant in the early stages of appendiceal inflammation. Several studies have shown that serum LRG1 levels rise significantly in patients with acute appendicitis compared to healthy controls and those with nonspecific abdominal pain [78,79]. Importantly, LRG1 exhibits good diagnostic accuracy, with sensitivity and specificity values that are comparable or superior to traditional markers like C-reactive protein (CRP) and white blood cell (WBC) count. Elevated LRG1 levels also correlate with the severity of appendiceal inflammation, suggesting its potential usefulness in distinguishing uncomplicated from complicated cases [78,79]. Unlike CRP, which often peaks later in the inflammatory process, LRG1 appears to increase earlier, making it useful for prompt diagnosis. Additionally, its relative stability in serum enhances its practicality for routine clinical testing. Despite these advantages, larger multicenter studies are needed to validate cutoff values and establish LRG1 as part of a standardized biomarker panel. Overall, LRG1 is a valuable addition to the growing landscape of serum biomarkers for acute appendicitis. Notably, LRG1 has also been detected in saliva, where early studies suggest a correlation with serum levels, opening the possibility of a non-invasive diagnostic tool in the future [78].

### 5.2. Urinary Biomarkers

Urinary biomarkers appear to be beneficial for children because they are non-invasive, painless, and simple to perform. Biomarkers like 5-hydroxyindoleacetic acid (5-HIAA) and leucine-rich alpha-2-glycoprotein (LRG) are being studied for their potential in diagnosing acute appendicitis, especially in children [80,81].

#### 5.2.1. 5-Hydroxyindoleacetic Acid (5-HIAA)

5-HIAA is a metabolite of serotonin that occurs in high concentrations in the appendix. Elevated levels of 5-HIAA in urine are linked to appendicitis, as the inflamed appendix releases more serotonin, which is then converted to 5-HIAA [82].

Although early studies showed promising results for 5-HIAA as a diagnostic tool, especially when combined with other clinical scores like the Paediatric Appendicitis Score (PAS), later research suggested it might not be as effective at ruling out appendicitis [83]. Some studies have found that 5-HIAA levels may be higher in the early stages of appendicitis and decrease in later stages, which makes it less useful for diagnosing advanced cases [80].

#### 5.2.2. Leucine-Rich Alpha-2-Glycoprotein (LRG)

A recent study introduced the “Appendicitis Urinary Biomarker (AuB) score,” which combines urinary LRG levels with three clinical variables to identify children at low risk of appendicitis [81,84]. LRG has demonstrated promising diagnostic accuracy, especially when used alongside the Paediatric Appendicitis Score, which exhibits high sensitivity, specificity, positive predictive value, and negative predictive value [81].

#### 5.2.3. Calprotectin

Calprotectin is a protein complex released by neutrophils during inflammation. Calprotectin is not a reliable urine biomarker for appendicitis, as studies show no significant differences in urine values between groups with and without appendicitis [85]. In contrast, salivary calprotectin has been examined in other inflammatory conditions, but its role in appendicitis remains unclear and requires further research.

#### 5.2.4. Serum Amyloid A (SAA)

The SAA is an acute-phase protein produced by the liver. SAA has shown its potential as a biomarker for early appendicitis, with circulating levels significantly increased in patients with this condition. While calprotectin is a recognized biomarker in other inflammatory diseases and in stool or serum for appendicitis, its use in urine is less established, whereas SAA is promising for appendicitis screening and diagnosis in blood [85]. Preliminary work also suggests that SAA can be detected in saliva, but evidence is very limited compared to serum, and further validation is needed to determine its usefulness.

## 6. Clinical Implementation and Practical Considerations

Despite the promising diagnostic performance of salivary biomarkers for pediatric appendicitis, especially in terms of specificity and correlation with serum values, their integration into clinical workflows remains limited [58,78]. This section outlines the practical, logistical, and ethical aspects that must be considered before routine clinical adoption.

Salivary biomarker testing may offer particular advantages in clinical contexts where conventional diagnostic tools are limited or yield inconclusive results. This approach is especially relevant for children who present with non-specific or atypical symptoms in primary care or emergency settings, as well as in environments where access to imaging modalities such as ultrasound or computed tomography is restricted [58,86,87,88]. Moreover, the rapid and non-invasive nature of salivary diagnostics renders them well suited to high-throughput pediatric emergency departments where efficient triage is essential [16,89].

Integrating salivary testing with established clinical scoring systems, such as the Pediatric Appendicitis Score or the Alvarado score, has the potential to enhance diagnostic confidence and facilitate more accurate stratification of patients for observation, further imaging, or surgical intervention [24,58].

### 6.1. Sample Collection and Analysis

Salivary sampling provides a convenient, non-invasive alternative to conventional specimens, with advantages of easy collection, minimal discomfort, and simple storage and transport. It is particularly useful in children, newborns, and individuals with bleeding disorders [90,91]. The stability of biomarkers such as CRP at room temperature further supports its feasibility in clinical and community settings [88].

Several collection methods exist, each with strengths and drawbacks. The most reliable is passive drool, where saliva accumulates and is guided into a tube; it is simple, requires no training, and can be automated with wearable devices [16,91,92]. Other approaches include spitting, chewing, and swabs. Spitting primarily samples submandibular and minor glands, while chewing (often with wax) stimulates parotid secretion [16,91]. Swabs, widely used during COVID-19, are especially suitable for children, though cotton swabs may alter biomarker concentrations [27]. Unstimulated and stimulated saliva also differ in flow rate, composition, and function [89].

Despite its promise, salivary diagnostics faces key challenges. Lack of standardized protocols for collection, handling, and storage introduces variability and limits reproducibility [88,93]. Pre-analytical factors, including circadian rhythms, fasting, oral hygiene, and recent food or fluid intake, can alter salivary composition [88,89,91,93]. These influences highlight the need for clear collection guidelines to control timing, patient preparation, flow rate, and dilution. Additionally, the absence of reference values hampers interpretation, and disease-specific biomarkers with validated sensitivity and specificity remain to be defined [88,91]. Addressing these gaps through standardization will be crucial to realizing saliva’s full diagnostic potential.

### 6.2. Integration into Point-of-Care Testing (POCT)

Early and accurate diagnosis is essential for timely medical intervention and optimal patient outcomes. While centralized, laboratory-based assays remain the dominant approach, there is growing pressure on healthcare systems to adopt non-invasive and accessible alternatives [16]. Saliva offers a promising diagnostic medium for POCT because it is non-invasive, painless, stable, and less complex than blood, while also being safer to handle and easier to collect in both clinical and non-clinical settings [94]. These advantages support its integration into portable, low-cost biosensors and dipstick-style lateral flow assays that could enable rapid screening in community and rural settings, pediatric outpatient clinics, pre-hospital care, or telemedicine-supported triage [86,90,91]. Salivary POCT offers a child-friendly, non-invasive alternative that enables rapid decision-making in emergency and primary care. By reducing discomfort and wait times, it supports earlier discharge, improves compliance, and expands access to diagnostics in resource-limited pediatric settings [95,96]. Early prototypes of salivary CRP and LRG1 lateral flow assays have demonstrated technical feasibility, though appendicitis-specific commercial products are not yet available [25,58,78,97]. Advances in biosensor technology hold potential to enhance salivary diagnostics, making them more accessible and applicable in real-time settings [86,90,98].

### 6.3. Cost and Resource Considerations

Salivary POCT has the potential to reduce healthcare costs by lowering the need for imaging, limiting unnecessary hospital admissions, and minimizing avoidable surgical explorations (trey). Rapid turnaround may also increase throughput in emergency settings and improve efficiency in overburdened hospitals. Peripheral and rural hospitals could benefit by using salivary POCT to diagnose uncomplicated cases locally while stabilizing and transferring critically ill patients to tertiary centers [95,96]. Nonetheless, initial development, validation, and regulatory approval of salivary POCT devices remain resource-intensive. Current salivary assays still depend largely on centralized laboratories, and the absence of standardized, widely available point-of-care platforms limits immediate clinical adoption [16,86,91]. Furthermore, health-economic evaluations will be essential to demonstrate the cost-effectiveness of large-scale implementation.

### 6.4. Ethical and Pediatric-Specific Benefits

Salivary diagnostics hold particular promise in pediatric care, where fear of needles and discomfort from invasive procedures often reduce compliance. Saliva collection is painless, non-invasive, and can be performed repeatedly without risk, which makes it especially suitable for children, neonates, and patients with coagulation disorders or immunocompromised conditions [90,99]. By minimizing procedural anxiety, salivary testing encourages earlier presentation, improves cooperation during testing, and aligns with principles of patient-centered and minimally invasive care. Beyond pediatrics, saliva’s ease of collection and reduced biohazard disposal requirements contribute to more sustainable healthcare practices [95]. Taken together, these advantages suggest that salivary diagnostics could serve as a useful complement to existing approaches, helping to improve accessibility, reduce costs, and enhance patient compliance, while supporting the broader goals of precision and preventative medicine.

### 6.5. Implications for Clinical Practice

Although evidence is still limited, salivary biomarkers show promise as adjunctive tools in the diagnostic workup of pediatric appendicitis, particularly when blood sampling is difficult or when imaging is inconclusive. Among investigated markers, salivary CRP has demonstrated the strongest performance and may support triage decisions in emergency settings. LRG1, despite low sensitivity, offers excellent specificity and could help confirm the diagnosis, while irisin may serve as a complementary screening marker.

In practice, saliva-based assays could be integrated with existing clinical scores or imaging algorithms to strengthen diagnostic confidence, reduce unnecessary radiation exposure, and guide timely surgical referral. With further validation and point-of-care development, salivary testing could become a feasible and child-friendly adjunct to current pathways.

## 7. Gaps in Literature and Limitations of Current Evidence

Despite the increasing interest in salivary biomarkers for diagnosing pediatric appendicitis, the current amount of evidence is very limited. So far only a few studies focused on saliva have been published, indicating that this research area is still at its beginning. Most of these studies are small-scale, single-centre, and exploratory or pilot in nature, which restricts their findings and elevates the risk of bias.

Another significant limitation is the lack of standardized saliva collection and handling protocols. Differences in sampling methods, pre-analytical processing, and storage conditions introduce variability and hinder reproducibility across studies. In addition, there is an absence of multicenter validation and longitudinal studies, which are essential to confirm diagnostic performance, assess temporal variations, and ensure clinical applicability.

Next limitation is the absence of multi-marker panels or the integration of omics-based approaches, which could offer a more comprehensive view of the salivary biomarker and enhance diagnostic accuracy. Additionally, no studies have performed validation or longitudinal analyses, leaving unresolved questions about reproducibility, temporal variations, and clinical applicability.

Furthermore, all existing research has concentrated solely on pediatric populations, with no studies examining adults. This creates a critical gap in understanding whether salivary biomarkers can be effectively utilized across different age groups. Collectively, these limitations highlight the need for larger, multi-center, and methodologically robust studies, including validation cohorts, longitudinal designs, and multi-omics strategies, to establish saliva as a dependable diagnostic tool for appendicitis.

## 8. Future Directions

Developing salivary biomarker panels that incorporate markers such as LRG1, CRP, IL-6, and selected microRNAs could enhance diagnostic accuracy compared to single-marker approaches.

Integrating these biomarkers into machine learning models and diagnostic algorithms presents a promising strategy to aid clinical decision-making, potentially minimizing unnecessary imaging and hospital admissions [100]. Additionally, efforts should focus on creating point-of-care biosensors or salivary dipsticks, allowing for rapid, non-invasive testing at the bedside.

Expanding research to include adult populations and conducting comparative studies will help assess the broader applicability of salivary diagnostics across different age groups. Lastly, it is essential to address ethical and regulatory considerations specific to pediatric point-of-care testing to ensure safe, equitable, and responsible implementation in clinical practice.

## 9. Conclusions

Salivary biomarkers for pediatric appendicitis offer a promising non-invasive diagnostic approach, but the field is still underexplored. Currently, the evidence is confined to just three pilot studies examining markers like LRG1, CRP, and irisin. To incorporate these biomarkers into routine clinical practice, it is essential to conduct more comprehensive, systematic, and multicentric research to validate their diagnostic accuracy and clinical utility.

## Figures and Tables

**Table 1:** Key findings from studies examining salivary biomarkers in pediatric appendicitis.

Study	Biomarker	Sample Size	Method	Findings	AUC	Sensitivity/Specificity	*p*	Notes
Yap et al., 2020 [56]	LRG1	34 (17 AA, 17 controls)	ELISA	Salivary LRG1 is elevated in AA; median significantly higher (294 ng/mL vs. 126 ng/mL)	0.770	35.3%/100% at cutoff value of >330 ng/mL	0.008	Pilot pediatric study; first to explore salivary LRG1 in appendicitis
Tintor et al., 2023 [21]	LRG1	92 (46 AA, 46 controls)	ELISA	LRG1 elevated in AA (median 233.5 ng/mL vs. 56.0)	0.853	36%/100% at cutoff value of >352.6 ng/mL	<0.0001	Largest salivary LRG1 study to date in pediatric appendicitis
Bakal et al., 2016 [57]	Irisin	60 (30 AA, 30 controls)	ELISA	Irisin is elevated in all fluids in AA; salivary data not analyzed separately for AUC	≈0.750 *	90%/60% at cutoff value of 19.6 ng/mL	<0.001	Exploratory study; no saliva-specific cutoff or AUC
Milunović et al., 2025 [58]	CRP	89 (46 AA, 43 controls)	ELISA	Salivary CRP is significantly elevated in AA (35.7 vs. 1.1 mg/L); strong correlation with serum CRP	0.970	91.3%/95.4% at cutoff value of >6.95 mg/L	<0.001	First salivary CRP study in pediatric appendicitis (Spearman ρ = 0.963)

AUC—area under the curve; AA—acute appendicitis; LRG1—leucine-rich α-2-glycoprotein 1; CRP—C-reactive protein; ELISA—enzyme-linked immunosorbent assay. * The AUC value was not explicitly reported by the authors but was estimated based on the ROC curve and the reported sensitivity and specificity values.

## Data Availability

Not applicable.
